# Leptin Overexpression as a Poor Prognostic Factor for Colorectal Cancer

**DOI:** 10.1155/2020/7532514

**Published:** 2020-06-03

**Authors:** Chunxiang Li, Jichuan Quan, Ran Wei, Zhixun Zhao, Xu Guan, Zheng Liu, Shuangmei Zou, Xishan Wang, Zheng Jiang

**Affiliations:** ^1^Department of Thoracic Surgery, National Cancer Center/National Clinical Research Center for Cancer/Cancer Hospital, Chinese Academy of Medical Sciences and Peking Union Medical College, Beijing 100020, China; ^2^Department of Colorectal Surgery, National Cancer Center/National Clinical Research Center for Cancer/Cancer Hospital, Chinese Academy of Medical Sciences and Peking Union Medical College, Beijing 100020, China; ^3^Department of Pathology, National Cancer Center/National Clinical Research Center for Cancer/Cancer Hospital, Chinese Academy of Medical Sciences and Peking Union Medical College, Beijing 100020, China

## Abstract

Leptin acts as an adipocytokine functions via the leptin receptor, which stimulates growth, migration, and invasion of cancer cells. This study is aimed at identifying leptin as a prognostic factor in colorectal cancer (CRC). The differentially expressed genes with prognostic value in CRC tissues either with or without liver metastasis were assessed based on The Cancer Genomic Atlas (TCGA). Leptin was considered a candidate gene for further analysis. Its expression features of 206 CRC patients without liver metastasis and 201 patients with metastasis on tissue microarrays were assessed by immunochemical staining, and the effect of leptin on survival was assessed by Kaplan-Meier analyses. Overexpressed leptin indicated a poorer prognosis for CRC patients in overall survival (*p* < 0.05, log-rank test) based on the TCGA database. The leptin expression significantly correlated with metastasis stage (*p* < .010) and lymph node involvement (*p* < .010). Multivariate analysis also indicated that strong leptin expression was an independent adverse prognosticator in CRC (*p* = .017). Leptin may be valued as a prognostic marker could contribute to predicting a clinical outcome for patients with CRC.

## 1. Introduction

Colorectal cancer (CRC) is one of the most common cancers worldwide. Approximately 20% of patients have distant metastatic disease at diagnosis [1]. Although 5-fluorouracil-based systemic therapy has markedly improved the outcome of patients with metastatic CRC, prognosis remains poor [2, 3]. Better understanding the clinically relevant molecular underpinnings will shed light on new paths for the prevention and even reversal of existing conditions.

As it stimulates several key pathways well known for their role in cell development, leptin has been classified as a growth factor [4], which is a product of the obesity gene. Growing evidence also suggests that it plays a key role in promoting tumor cell proliferation and migration [5]. Leptin, as a well-established risk factor for individual cancers [6], is attracting the focus of more research. Recent evidence indicated that leptin promotes breast cancer metastasis by activating the JAK/STAT3 and PI3K/AKT signaling pathways [7], which is consistent with the results on CRC [8].

Several clinical studies have shown the clinical significance of leptin in CRC [9–14], however, the results remained inconsistent. It might be due to a small sample size and different patient population. Hence, a study with rigorous methods is highly necessary to perform. The aim of this study is to assess the impact of leptin on CRC, which assists in early identification of high-risk patients.

## 2. Materials and Methods

### 2.1. Bioinformatics Analysis

Gene expression data and clinical information from COAD projects (490 cases, workflow type: HTSeq-counts) were collected from TCGA. Normal COAD, overall survival < 30 days, and unavailable or unknown clinical features samples were excluded. Finally, the data contains 312 samples of M0 colorectal cancer tissues, and 50 samples of M1 colorectal cancer tissues were used for further analyses.

The Database for Annotation, Visualization and Integrated Discovery (DAVID, https://david.ncifcrf.gov/) v6.8 is utilized to assess the functional level of different expression genes (DEGs). These gene functional enrichment analyses were demonstrated with using the clusterProfiler package of R. When the inclusion significantly different was normalized *p* < 0.05, the analysis of gene functional pathway was treated as being significantly enriched by these genes. The gene ontology (GO) plot package of R software was used to perform the results of the GO and Kyoto Encyclopedia of Genes and Genomes (KEGG) analyses.

The Retrieval of Interacting Genes (STRING) database (http://www.string-db.org/) was performed to assess the data of protein–protein interaction (PPI) network and potential relationships among DEGs, which could be mapped in Cytoscape software (version 3.7.1). To identify the hub of genes in each module that is based on the score of each gene in the module, the cytoHubba plugin was utilized for searching the hub genes from the network [15]. EcCentricity was used for the cytoHubba criteria for calculating nodes' scores.

Gene set enrichment analysis (GSEA) (http://www.broadinstitute.org/gsea/index.jsp) was utilized to investigate the mechanisms related to leptin expression in CRC patients. The 362 CRC samples in TCGA-COAD were grouped into the high- and low-expression groups by the median leptin expression. One thousand permutations for gene sampling were utilized to verify statistical significance and ensure the guarantee of the results. The inclusion significantly different was normalized *p* < 0.05 and false discovery rate (FDR) < 25%.

We explore the significance of hub genes among colorectal cancer. Clinical data and mRNA expression in the COAD (TCGA) database were obtained from the TCGA online platform. CRC patients were figured into analysis to assess the relationship between hub genes and survival by using the median cutoff of mRNA expression.

### 2.2. Patients and Tissue Samples

The specimens were collected from the patients who received operation between 2006 and 2012 at our center. The inclusive criteria were as follows: (a) without neoadjuvant radiotherapy or chemotherapy and (b) with complete clinical and follow-up information. Totally, 206 CRC cases without liver metastasis and 201 cases with simultaneous liver metastasis were collected based on the inclusive criteria. All patients provided a written informed consent, and the research protocol was approved by the ethics committee at our center that approved this study (NCC2016JZ-06). All patients were followed up every three months until 31 December 2017.

### 2.3. Tissue Microarray (TMA) and Immunohistochemistry

The CRC without metastasis cohort included the tumor and normal tissue of each patient, and CRC with liver metastasis cohort consisted of the normal intestinal mucosa, primary tumor, metastatic tumor, and normal liver tissue from each patient. The TMAs were built as described previously [16].

Immunohistochemical staining was done on the slides (5 *μ*m thick) from the TMAs, using a Leptin rabbit monoclonal antibody (1 : 2000; Cell Signaling Technology, United States). Cytoplasm staining was measured for this antibody. The slides were reviewed by two pathologists independently who were blind to the clinical outcome. A 4-tiered scoring system (negative to 3+) was used for the clinicopathological correlation as described previously [17].

### 2.4. Statistical Analysis

SPSS version 13.0 is used. The Mann–Whitney *U* test or the Kruskal-Wallis *H* test is used to examine the difference between groups. Intergroup difference was compared using the Chi-square test or Fisher exact test. Survival rate was plotted using the Kaplan-Meier method and analyzed using the log-rank test. Multivariate analysis was done by COX proportional hazard model. A value of *p* < 0.05 was considered significant.

## 3. Results

### 3.1. Leptin Was Selected as a Candidate Gene for Further Analysis

**Figure 1:**
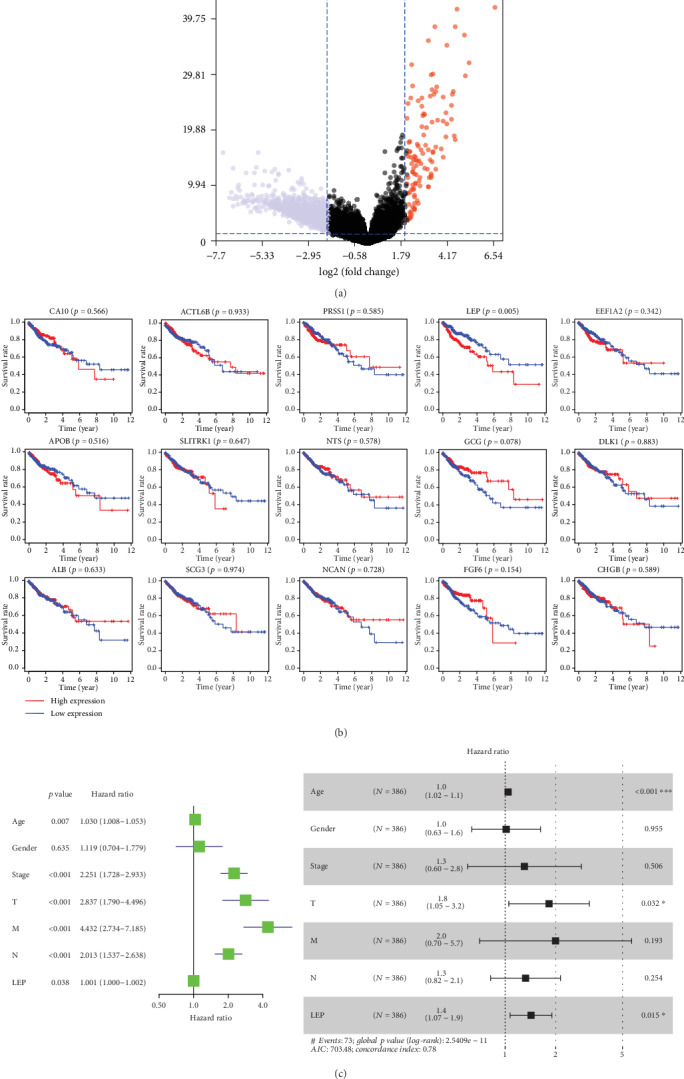
(a) The volcano plot of differentially expressed genes of M0 and M1 CRC tissues. (b) Survival analysis for the hub genes. The Kaplan-Meier test between hub genes high-expression and low-expression patients. (c) Univariate and multi-Cox analysis of leptin expression are associated with OS in colorectal cancer patients.

On the basis of the selection criteria, the differentially expressed genes of M0 and M1 from TCGA were analyzed and screened with software R (version 3.4.1). A total of 738 differentially expressed genes were identified and an adjusted *p* value < 0.05 and ∣log2FC (foldchange) | ≥2 are used. The result is shown in the volcano plot in Figure 1. GO and KEGG analyses were applied to determine the roles of these differentially expressed genes, as shown in Figure S1. Utilizing the information from the STRING database, a PPI network for the 738 genes consisting of 167 nodes and 1178 edges was constructed using Cytoscape software (version 3.7.1). To further analyze the hub genes in the PPI network, EcCentricity of cytoHubba plugin was used to extract the following top 15 genes as hub genes: SCG3, APOB, DLK1, FGF6, GCG, PRSS1, NTS, CA10, LEP, ALB, ACTL6B, SLITRK1, EEF1A2, NCAN, and CHGB (Figure S2). Matching the clinical data with the TCGA mRNA profile, 362 CRC samples were grouped by the median cutoff of the mRNA expression levels of 15 genes. And only a high level of leptin mRNA expression is correlated with poor overall survival (*p* < 0.005, Figure 1).

The potential risk factors were analyzed utilizing the Cox regression analysis. Univariate analysis revealed that upregulated leptin was a significant risk factor associated with OS for CRC patients (all *p* < 0 05, Figure 1). And Cox regression analysis showed that the upregulated leptin's age and T stage were significant risk factors (*p* < 0 05, Figure 1). To explore the function of leptin, GSEA was performed in TCGA-COAD. The samples in the leptin overexpression group were enriched in cell cycle-, focal adhesion-, oxidative phosphorylation-, and oncogene-related functions (Figure S3).

### 3.2. Correlation between Leptin Expression and Clinicopathological Features

**Figure 2:**
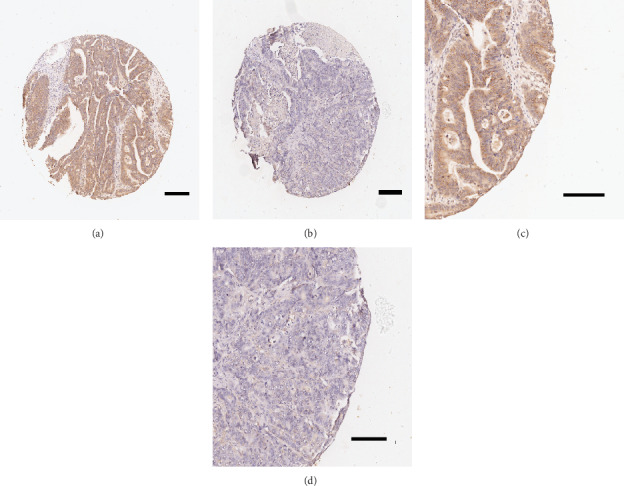
Immunohistochemistry staining showing leptin expression in CRC tissues. Tissue high expression (4x for (a), 10x for (c)) and low expression (4x for (b), 10x for (d)) for the leptin protein are shown. Each of the punched samples is 1.0 mm in length in the tissue microarrays.

To validate the foregoing results, we assessed the leptin expression patterns in the CRC cohort and liver metastasis cohort. Morphologically, leptin was primarily localized in the cytoplasm of the cancer cells. Immunostaining of leptin protein was low (- and 1+) in some samples but strong (2+ and 3+; Figure 2) in others. It revealed that 52% of samples demonstrate strong intensities and 48% low intensities.

No significant differences regarding histologic grading, T stage, and adjuvant therapy were found. However, lymph node involvement was significantly different between patients with strong leptin protein expression and those with low expression (*p* < .010). Moreover, the leptin expression was significantly associated with M stage (*p* < .010).  Table 1 displayed the relationship between clinicopathological features and leptin protein expression in 407 samples.

### 3.3. The Expression of Leptin Is Associated with Survival Outcome

**Figure 3:**
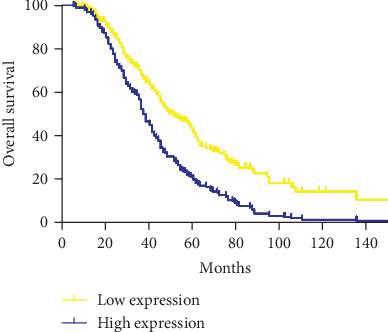
Kaplan-Meier curve of patients in the strong leptin expresser and the low ones.

It revealed that leptin overexpression was significantly associated with unfavorable OS by Kaplan-Meier survival analysis (*p* = .0014, log-rank test). Also, the significantly different survival distributions were observed in CRC patients according to M stage and lymph node involvement. A characteristic plot is displayed in Figure 3. However, the clinicopathologic factors such as tumor location, differentiation grade, T stage, preoperative CEA level, and microsatellite instability (MSI) status had no significant correlation with the OS. M stage (*p* < .01) and leptin expression (*p* = .017) were identified as independent variables by multivariate Cox analysis. The distant metastasis and strong leptin expression were adverse factors of prognosis (Table 2).

## 4. Discussion

The prognosis of patients with CRC is determined by recurrence status after resection of the primary tumor. More than one-half of the patients undergoing resection of primary CRC have recurrence, mostly in the liver and the lung [18]. For CRC metastases in the liver, there is no curative chemotherapeutic treatment available. Resection is a therapy of choice for the treatment of single metastatic nodules. Unfortunately, the vast majority of patients are deemed unsuitable for surgical resection at initial diagnosis [19, 20]. Therefore, we need to identify more relevant prognostic factors for selecting patients at high-risk of metastasis from CRC likely to benefit from treatment.

In this study, we assessed gene microarray in the setting of CRC liver metastasis from the TCGA, which is aimed at detecting the leptin expression pattern between CRC primary samples with and without liver metastasis. The role of leptin in CRC based on TCGA was also assessed. Subsequently, we further measured the expression pattern by immunohistochemistry and examined the related clinicopathological characteristics and survival outcomes. When we looked at the differences in the expression between primary tumors with and without liver metastases, the expression levels of leptin progressively increased from early-stage to late-stage disease. Previous studies have found that leptin is a prognostic marker [13]. Thus, this consistent increase of expression in CRCs with liver metastasis likely represents the generally poor prognosis of cancers which mostly progress to advanced disease.

Leptin acts as a growth factor in colon epithelial cells. Binding of leptin to its receptor activates transcription (JAK/STAT) and Ras/extracellular signal-regulated kinase signal transduction pathways [21], inducing tumor cell proliferation and migration and suppressing apoptosis. However, previous findings regarding leptin expression and CRC risk have been conflicting. It could be due to sample size, diversity of populations, semiquantitative staining protocol, and procedure sensitivity [9]. To address these shortcomings, we obtained and analyzed the required data in the TCGA database and validated the results analyzed by a cohort with a relatively large sample size, which is a key strength of this study.

In conclusion, our study investigated the role of leptin in CRC using a rigorous scientific approach. It revealed that leptin overexpression is an independent indicator for an adverse prognosis, which might contribute to identifying high-risk surgical patients with CRC.

## Figures and Tables

**Table 1:** Relationships between leptin expression and the clinical profiles of CRC.

Factor	*N* (%)	Leptin expression		*p* value
		Strong	Low	
Age				
≥65	91	45	46	
<65	316	165	151	0.73
Gender				
Male	236	127	109	
Female	171	93	78	0.99
Tumor location				
Colon	189	94	95	
Rectum	218	116	102	0.55
Histologic grading				
Low grade, moderate grade	365	192	173	
High grade	42	18	24	0.30
Preoperative CEA level (ng/mL)				
≤5	181	97	84	
>5	226	113	113	0.53
Lymph node involvement				
Yes	197	142	55	<0.01
No	210	68	142	
T stage				
T3	128	74	54	
T4	279	136	143	0.11
M stage				
M0	206	87	119	
M1	201	123	78	<0.01
Adjuvant therapy				
Yes	374	192	182	
No	33	18	15	0.86
MSI				
MSS/MSI-L	372	194	178	
MSI-H	35	16	19	0.58

CEA: carcinoembryonic antigen; MSI: microsatellite instability; MSS: microsatellite stable; MSI-L: microsatellite instability-low; MSI-H: microsatellite instability-high; T stage: tumor stage; M stage: distant metastasis.

**Table 2:** Cox analyses of potential prognostic factors for overall survival in CRC.

Factor	Comparison	Univariate analysis			Multivariate analysis		
		HR	95% CI	*p* value	HR	95% CI	*p* value
Lymph node involvement	Yes vs. no	1.649	1.068-2.369	0.021	1.423	0.976-1.743	0.053
M stage	M1 vs. M0	2.137	1.936-4.312	0.004	2.437	1.692-3.175	0.002
Leptin expression	High vs. low	2.034	1.912-3.425	0.007	2.011	1.279-2.231	0.017

HR: hazard ratio; CI: confidence interval.

## Data Availability

The TCGA data used in this study was downloaded from the Genomic Data Commons Data Portal https://portal.gdc.cancer.gov/ and the validation cohort data is provided as the Supplementary Dataset.

## Supplementary Materials

Supplementary Materials (1) Supplementary Figure 1: functional enrichment analysis of overlapped differentially expressed genes of M0 and M1 CRC tissues. (A) GO enrichment analysis of overlapped differentially expressed genes in BP. (B) GO enrichment analysis of overlapped differentially expressed genes in MF. (C) GO enrichment analysis of overlapped differentially expressed genes in CC. (D) KEGG analysis of overlapped differentially expressed genes. (2) Supplementary Figure 2: the PPI network is made up of the 738 overlapped differentially expressed genes. (A) The PPI network of the overlapped differentially expressed genes. (B) The PPI network of the major part of hub genes. (3) Supplementary Figure 3: gene set enrichment analysis for the high-leptin expression group and low-leptin expression group. (4) Supplementary data: the validation data used in this study.
