# Supplementary material for: Leptin Overexpression as a Poor Prognostic Factor for Colorectal Cancer
Source: Biomed Res Int. 2020 Jun 3;2020:7532514. doi: 10.1155/2020/7532514 (PMC7292990; doi:10.1155/2020/7532514)
Supplement: Supplementary Materials — (1) Supplementary Figure 1: functional enrichment analysis of overlapped differentially expressed genes of M0 and M1 CRC tissues. (A) GO enrichment analysis of overlapped differentially expressed genes in BP. (B) GO enrichment analysis of overlapped differentially expressed genes in MF. (C) GO enrichment analysis of overlapped differentially expressed genes in CC. (D) KEGG analysis of overlapped differentially expressed genes. (2) Supplementary Figure 2: the PPI network is made up of the 738 overlapped differentially expressed genes. (A) The PPI network of the overlapped differentially expressed genes. (B) The PPI network of the major part of hub genes. (3) Supplementary Figure 3: gene set enrichment analysis for the high-leptin expression group and low-leptin expression group. (4) Supplementary data: the validation data used in this study. [file 7532514.f1.pdf]

Supplementary Materials

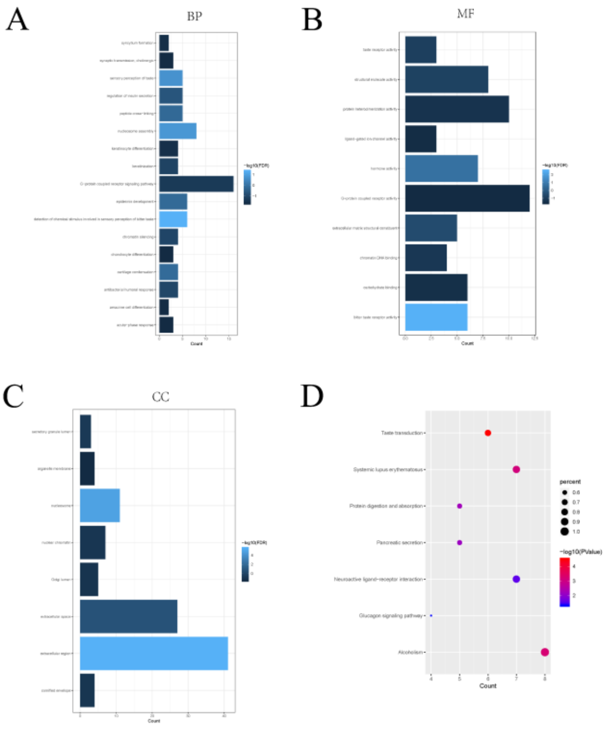

Figure S1. Functional enrichment analysis of overlapped differentially expressed genes of M0 and M1 CRC tissues. (A) GO enrichment analysis of overlapped differentially expressed genes in BP; (B) GO enrichment analysis of overlapped differentially expressed genes in MF; (C) GO enrichment analysis of overlapped differentially expressed genes in CC (D) KEGG analysis of overlapped differentially expressed genes.

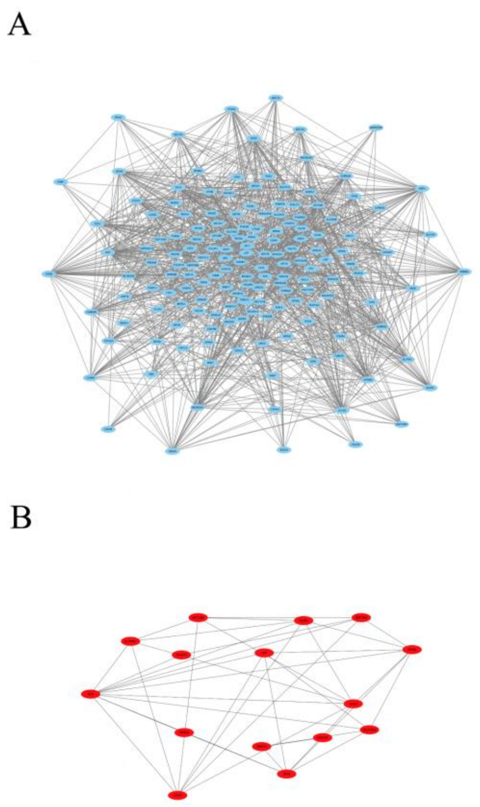

Figure S2. The PPI network is made up of the 738 overlapped differentially expressed genes. (A) The PPI network of the overlapped differentially expressed genes; (B) The PPI network of the major part of hub genes.

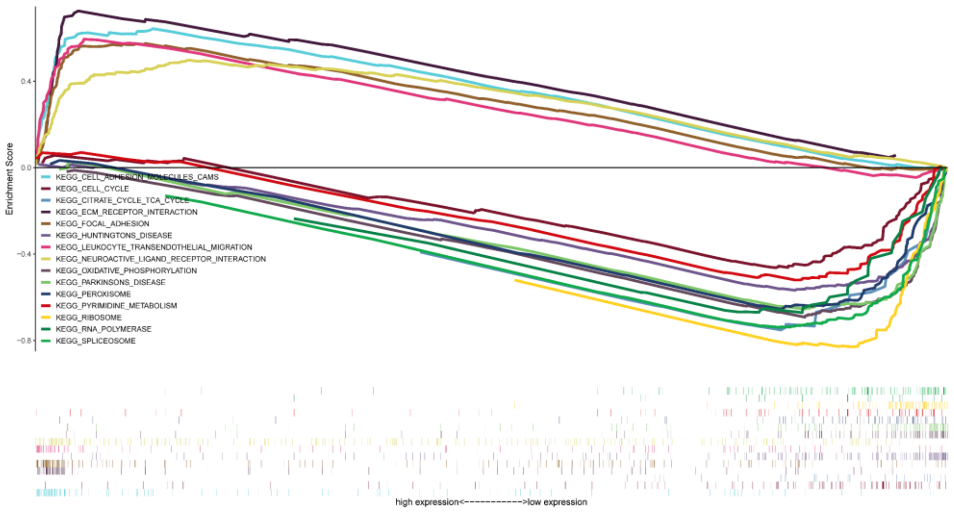

Figure S3. Gene set enrichment analysis for high leptin expression group and low leptin expression group.
